# The Potential Role of Creatine in Vascular Health

**DOI:** 10.3390/nu13030857

**Published:** 2021-03-05

**Authors:** Holly Clarke, Robert C. Hickner, Michael J. Ormsbee

**Affiliations:** 1 Department of Nutrition, Food and Exercise Sciences, Florida State University, Tallahassee, FL 32306, USA; hec17e@my.fsu.edu (H.C.); rhickner@fsu.edu (R.C.H.); 2 Department of Biokenetics, Exercise and Leisure Sciences, School of Health Science, University of KwaZulu-Natal, Westville 4041, South Africa; 3 Institute of Sports Sciences and Medicine, Florida State University, 1104 Spirit Way, Tallahassee, FL 32306, USA

**Keywords:** creatine, vascular pathology, cardiovascular disease, oxidative stress, vascular health

## Abstract

Creatine is an organic compound, consumed exogenously in the diet and synthesized endogenously via an intricate inter-organ process. Functioning in conjunction with creatine kinase, creatine has long been known for its pivotal role in cellular energy provision and energy shuttling. In addition to the abundance of evidence supporting the ergogenic benefits of creatine supplementation, recent evidence suggests a far broader application for creatine within various myopathies, neurodegenerative diseases, and other pathologies. Furthermore, creatine has been found to exhibit non-energy related properties, contributing as a possible direct and in-direct antioxidant and eliciting anti-inflammatory effects. In spite of the new clinical success of supplemental creatine, there is little scientific insight into the potential effects of creatine on cardiovascular disease (CVD), the leading cause of mortality. Taking into consideration the non-energy related actions of creatine, highlighted in this review, it can be speculated that creatine supplementation may serve as an adjuvant therapy for the management of vascular health in at-risk populations. This review, therefore, not only aims to summarize the current literature surrounding creatine and vascular health, but to also shed light onto the potential mechanisms in which creatine may be able to serve as a beneficial supplement capable of imparting vascular-protective properties and promoting vascular health.

## 1. Introduction

According to the World Health Organization (WHO), since the 1970s, cardiovascular disease (CVD) has steadily remained the leading cause of mortality in developed countries around the world, taking an estimated 17.9 million lives every year. Within the United States (US) alone, it has been estimated that around 655,000 deaths occur annually due to CVDs [1]. The American Heart Association (AHA) reported that more than 43.7 million adults aged >60 years suffered from at least one or more CVD in 2016, with around two thirds of CVD deaths occurring in those aged >75 years [2]. Furthermore, the AHA predicts that without effective intervention 40% of US adults will have one or more CVDs by 2030 [3], placing a substantial financial strain on the healthcare system. Not forgoing the economic impact imparted by the 2020 worldwide coronavirus pandemic, the national healthcare expenditure projections to account for CVDs alone has been estimated to further rise to ~USD 5.7 trillion by 2026 [4]. Furthermore, CVD additionally has a major impact upon wellbeing and individual quality of life, with the incidence of CVD being closely associated with an increased risk of depression [5,6] and the reduction in ability to perform activities of daily living [7]. Considering the multidisciplinary impact of CVD, effective interventions and therapies are clearly warranted. In an attempt to avoid the reliance upon pharmaceuticals and often invasive surgical procedures, naturally derived therapies have grown in popularity for the prevention and management of CVD. Common examples can include nutritional choices such as the ingestion of nutrient dense, antioxidant rich “super foods” or dietary supplements. While multivitamins, fruits and vegetables are commonly praised for their contributions to health, there is growing evidence that suggests creatine supplementation may also serve as a potential nutritional adjuvant therapy [8,9]. This naturally occurring amino-acid derivative, often taken for its benefits to skeletal muscle, has been shown to serve as an anti-inflammatory and an antioxidant, among other advantageous properties [9]. These properties suggest creatine has potential to attenuate the detrimental characteristics of CVD.

CVD is an umbrella term used to denote a variety of pathological disorders of the heart and or the vessels that stem through the body and vascularize all tissues and organs. Although both the heart and vessels work collectively to function as the cardiovascular system, pathophysiological changes in either the heart or vasculature can independently contribute to the development of CVD. Common vascular-specific pathologies can include coronary artery disease, stroke, atherosclerosis, hypertension, and peripheral artery disease. Despite all being classified under a common umbrella term, the pathological etiology of each is multifactorial, with numerous risk factors contributing to the development and severity of each. Major examples of well recognized risk factors include dyslipidemia, hypertension, systemic inflammation, obesity, diabetes, tobacco use, lack of physical activity, and alcohol abuse; all of which represent more than 90% of the CVD risks in current epidemiological studies [10]. Physiologically speaking, vascular pathologies are commonly characterized by deteriorations in vascular integrity or alterations in vessel structure. Vessel walls can be described as consisting of three structurally distinct layers, or tunicas [11]. The tunica intima is the inner most layer lining the entire vascular system, consisting of flattened endothelial cells (ECs) arranged in a longitudinal manner in the direction of blood flow [11]. The tunica media provides vasotone, mechanical strength and contractile power, by which these properties are provided by the presence of vascular smooth muscle cells (VSMCs), embedded in an intricate network of elastin and collagen [12]. Finally, the tunica adventitia is the outermost layer and provides structure whilst anchoring vessels to surrounding tissues [11]. All vessels, with the exception of the capillaries found in the microvasculature, have varying degrees of each layer in accordance to their function and location. Any disturbance in these layers however can lead to cellular dysfunction and consequential challenges to vessel health and the development of vascular pathologies [13]. Some of the most common deteriorations seen in vessel structure include arterial wall thickening, wall enlargement, arterial stiffening, endothelial dysfunction, and inflammation [14,15,16,17]; all of which are further augmented by the risk factors previously mentioned.

It can be confidently concluded that the maintenance of healthy vasculature is paramount for longevity, which is in part why vascular pathology remains the leading cause of mortality in the world. Maintenance of the inner most ECs, found lining the vessels of the vascular system, has been found to be integral to overall vascular health and reduced risk of future adverse cardiovascular events [18,19,20]. ECs contribute to the delicate control and homeostasis of the vascular system, adapting to hormonal [21], mechanical [22], and neural stimuli [23] to sustain vascular function. ECs additionally contribute to fluid filtration, control of vasomotor tone via the release and synthesis of vasoactive factors such as nitric oxide (NO), regulation of both local and global blood flow, regulation of blood pressure, hemostasis, hormone trafficking, angiogenesis, immune response and inflammation [17,24]. Despite their complex contribution to vascular health, ECs are delicate and can be easily damaged by varying factors such as free radicals or reaction oxygen species (ROS) [25] and chronic inflammation [26], both of which can be augmented by, or a result of, risk factors such as dyslipidemia [27], hyperglycemia [28], tobacco use [29], alcohol abuse [30], obesity [31], and unfortunately the inevitable process of aging [32]. Any damage suffered can result in adverse alterations to endothelial physiology, consequentially leading to endothelial dysfunction (ED). ED can be characterized by a reduction in the bioavailability of NO, a potent vasoactive compound, which leads to the impairment of endothelium-dependent vasodilation often seen underlining many, if not all, CVDs [15]. Furthermore, ED often leads to ECs frequently expressing and releasing more procoagulation factors, shifting what would be a hemostatic balance towards a more prothrombotic and proinflammatory state [17], all of which similarly underly a multitude of CVDs [33]. 

## 2. Combatting the Development of Vascular Pathology 

While modern advances in medicine and pharmaceuticals such as blood thinners (anticoagulants), angiotensin-converting enzyme (ACE) inhibitors, beta-blockers, and diuretics have been shown to be effective for the management of vascular pathologies and CVDs, these are not without their limitations. Many medications come with inherent risk of side effects such as dizziness, constipation or diarrhea, skin irritations and excessive bleeding. These side effects, in addition to the financial costs commonly associated with prescriptions, often hinders the medical adherence of many consumers. A Keiser Family Foundation data note published in 2019 regarding prescription drugs in older adults, reported that 76% of older adults believe prescription medication cost is unreasonable, with 23% reporting difficulty in affording their prescribed medications [34]. Perhaps more shockingly, about one in five older adults (21%) reported not taking their medication as prescribed due to costs, with over half of these individuals not even informing their doctor or health care provider. Taking this data into consideration, despite the ability of pharmaceuticals to help manage the pathological characteristics of vascular diseases, it could be argued that other, or adjuvant, interventional strategies are comparably important to offer those at who are at risk of, or suffering from, vascular diseases or CVDs in general. 

With the noted limitations of pharmaceuticals, the consumption of dietary nutritional supplements, or the use of nutraceuticals or functional “super” foods, has increasingly grown in popularity and is utilized by many individuals to help promote health and wellbeing [35]. In the Council for Responsible Nutrition 2018 consumer survey, 75% of US adults reported taking some form of dietary supplement to either benefit overall wellness, fill nutrient gaps, promote bone health, benefit heart health, support healthy aging, and or to aid in joint health [35]. Some common “superfoods” reporting significant benefits to cardiovascular health include fruits and vegetables high in vitamin C, polyphenols, carotenoids and lycopene (citrus fruits, blueberries, red peppers, melon, strawberries, carrots, tomatoes), nuts and peanuts (walnuts, almonds), and whole grains [36]. Other dietary supplements applauded for their vascular benefits include l-arginine [37,38], co-enzyme Q10 [39,40], nitrites [41], tetrahydrobiopterin (BH4) [42], and l-citrulline [43]. Literature suggests that it is the vitamin richness, nutrient density, and antioxidant capacity of many of these that contribute to the benefits often reported [36,44,45,46]. 

Creatine is known as an efficacious and widely popular ergogenic supplement, often taken to help enhance energy stores and the buffering capacity of skeletal muscle during high-intensity exercises. Although a popular supplement, creatine can be found naturally in meat and fish sources, with creatine content ranging between 3–5 g/kg of raw meat [47]. Despite being found naturally, to achieve the typical 20 g/day “loading” amount of creatine necessary to quickly increase skeletal muscle stores [48,49], one would have to consume approximately 4 kg of meat per day. This high meat intake, for some, could be difficult to achieve due to dietary concerns, high calorie intake, cost, or other concerns such as the inherent high fat intake. Due to these factors, creatine is now synthetically produced and remains one of the most commonly consumed supplements [50]. Creatine monohydrate was one of the earliest forms of synthetic creatine, first marketed in 1990s, and remains the most commonly consumed and utilized form of creatine in scientific literature [50]. Other forms of creatine however have been marketed with varying claims of superior absorption, chemical and physiological properties, although these claims are often unsupported [50]. Examples of other marketed creatine analogs include creatine ethyl ester, creatine malate, creatine pyruvate, and sodium creatine phosphate; all of which have a lower creatine content percent than creatine monohydrate [50]. For the sake of this review, when discussing varying studies, the term “supplemental creatine” will denote the investigator’s use of creatine monohydrate. The use of another form, if used, will be noted where necessary. In addition to the known ergogenic benefits of creatine monohydrate [49,51], recent evidence also supports the ability of creatine to exhibit other non-energy related benefits, aiding in the attenuation of risk factors that are associated with vascular pathology. As aforementioned earlier, the accumulation of ROS is closely associated with a deterioration in EC health and vascular integrity. Although primarily in vitro data, there is evidence to suggest that creatine may serve as both a direct- and indirect-antioxidant [52,53], which could benefit vascular and EC health through ROS reduction. Furthermore, creatine has also been shown to serve as an anti-inflammatory agent [54], reducing EC damage induced by chronic and exercise induced inflammation. In addition to these, there is also evidence to suggest that creatine can help manage dyslipidemia [55,56], improve glycemic control [57], and improve mitochondrial function [58], all of which have been found to characterize vascular pathologies. It is due to, in part, these non-energy related properties that researchers believe is why creatine has shown such promise in other clinical populations such as those suffering from metabolic [57,59], muscular [60,61] and neurological diseases [62,63,64]. In addition to all the reported beneficial and pleiotropic properties of creatine, creatine supplementation also exhibits an excellent safety profile, with minimal reported side effects after acute or chronic supplementation, in moderate or large doses, in a variety of populations from young to old [49,65]. 

Despite the evidence supporting creatine’s application as an ergogenic aid, as a potential adjuvant role in the management of varying pathologies, and its ability to influence other disease risk factors, there is scarce scientific insight into the possible application of creatine for the improvement of vascular health and CVD. Taking into consideration that creatine has been found to elicit non-energy related benefits, such as reducing damaging free radicals and ROS, reducing inflammation, reducing mitochondria-specific ROS, and possibly reducing circulating levels of homocysteine [56,66], all of which have been scientifically linked closely to vascular disease [67,68,69], there may be mechanisms by which creatine could potentially impact vascular health. Furthermore, the lack of clinical trials in this area highlights a major gap in the creatine literature. Although the exact mechanisms by which creatine exerts these non-energy-related benefits are still relatively speculative, with CVD remaining the leading cause of mortality and the clear untapped potential of creatine, there is a need for further research and insight into the more novel applications and functions of creatine supplementation. 

The following sections of this review aim to outline the current literature available regarding creatine’s application for vascular health and function, in addition to potential mechanisms by which creatine may contribute towards vascular health. 

## 3. Existing Research on the Effects of Creatine Supplementation and Vascular Health

Since its isolation and extraction from skeletal muscle in 1832 by French chemist Michel Eugène Chevreul, the metabolic, ergogenic and physiological application of creatine has been extensively researched. Despite the recently found exciting potential of creatine to serve as an adjuvant therapy in varying clinical applications, in addition to the variety of non-energy-related properties that collectively highlight the expansive value of creatine, there have been few studies investigating the role of creatine for vascular health. Following an extensive literature search, we found only four clinical studies that reported investigating the direct impact of creatine supplementation on vascular health and function. Two of these studies [70,71] primarily looked at creatine’s impact upon the macrovasculature, specifically pertaining to the larger blood vessels such as the aorta and sizeable arteries in the brain and limbs; whereas, the remaining two studies [55,56] looked primarily at the impact of creatine on the microvasculature, the portion of the vascular system that is composed of the smallest vessels such as the arterioles and capillaries.

### 3.1. Creatine and the Macrovasculature 

Arciero et al. [70] was among the first to investigate the impact of creatine, taken either alone or in combination with resistance training, on blood flow of the lower leg (calf) and forearm. This randomized, double-blind, placebo-controlled study allocated 30 healthy male participants into one of three major groups: creatine only, creatine + resistance training, and placebo + resistance training. Both creatine and placebo (dextrose) supplementation protocols consisted of 20 g/day for the first 5 days, followed by 23 days of 10 g/day. To determine the resulting impact of supplement and/or training on blood flow, Arciero utilized the method of venous occlusion plethysmography, in which changes in limb circumference in response to rapid occlusion and reperfusion are indicative of limb blood flow, calculated as mL‧100 mL^−1^·min^−1^. Following creatine supplementation, the authors reported a significant increase in both calf and forearm blood flow; however, these changes were seen only in the creatine + resistance training group and not in the creatine alone or placebo group. Considering that effects were only significant when supplementation was taken in combination with resistance training, the authors concluded that these novel results indicate a “synergistic” interplay, or “additive” effect. Although these findings support those of other studies, indicating that the benefits of supplements are often augmented when used in combination with other physical interventions [72,73], Arciero’s reported findings when taken alone provide no definitive evidence to suggest that creatine could independently benefit vascular health. 

Similarly, in a double-blind placebo-controlled study, Sanchez-Gonzalez et al. [71] looked to determine the effect of three weeks of creatine supplementation (2 × 5 g/day), in comparison to a maltodextrin placebo, on hemodynamic and arterial stiffness responses after acute isokinetic exercise in 16 healthy young males. To determine hemodynamic responses, heart rate was monitored continuously using bipolar electrocardiogram (ECG), and arterial stiffness was assessed using the current non-invasive gold standard method, pulse wave velocity (PWV) [74]. Authors reported that following the 21-day supplement period, creatine attenuated the increase in systolic blood pressure (SBP) 5 min post-exercise, and heart rate response at both 5- and 15-min post-exercise. It was also reported that creatine suppressed the increase in brachial-ankle PWV (baPWV) following the fatiguing bout of isokinetic exercise, and that those in the creatine group displayed a faster return of heart rate to resting values in comparison to those in the placebo group. The authors concluded that creatine supplementation contributed to improved hemodynamic and vascular responses to acute isokinetic bouts of exercise. Although no direct mechanisms were assessed, this effect was speculated to be due to a reduction in left ventricle afterload and reduced muscle ammonia and lactic acid production, which would have otherwise led to sympathetic-mediated increases in heart rate and blood pressures [75]. As previously mentioned, increases in arterial stiffness often indicate the presence of impaired arterial wall health and is a major contributor to the development of CVD; therefore, these results suggest a potential benefit of creatine supplementation for vascular health. Furthermore, heart rate recovery time following exercise is a powerful indicator of overall mortality [76], again suggesting a benefit of creatine to cardiovascular health. These findings are in contrast to that of Arciero et al. 

### 3.2. Creatine and the Microvasculature 

Moraes et al. [55] was the first to investigate the impact of creatine supplementation on the systemic microcirculation, rather than macrocirculation. Moraes investigated the effect of one week of 20 g/day micronized creatine, a commonly utilized “loading” protocol [49,77], on systemic microcirculation, microvascular reactivity, and skin capillary density in young healthy males. In addition to microvascular assessments, Moraes also reported on the impact of creatine on blood lipids such as low-density lipoprotein cholesterol (LDL-C), high-density lipoprotein cholesterol (HDL-C) and total cholesterol, all of which have documented associations with CVD risk [78]. Furthermore, circulating homocysteine levels were assessed, a sulfhydryl-containing amino acid known to adversely impact vascular health and increase CVD risk [79]. Following supplementation, authors reported no significant impact upon circulating homocysteine levels; however, a significant reduction was seen in both total cholesterol and low-density lipoprotein cholesterol (LDL-C). It was further reported that creatine significantly increased skin functional capillary density and recruitment post-occlusive reactive hyperemia, and that cutaneous microvascular vasodilation induced by hyperemia also increased. Despite being extremely novel at the time, this study was not without its limitations. For example, this study lacked a true control group or placebo, was open label in that all participants were aware of the supplement they were receiving, and assessed variables following a relatively short time frame of creatine supplementation. Despite these noted limitations however, the improvements found by Moraes hold great promise and suggest potential vascular-protective properties for creatine. As mentioned previously in this review, the functional ability of ECs to synthesize and release potent vasoactive compounds to control vasomotor tone is vital for the management of blood flow, blood distribution, and blood pressures. The inability of ECs to release vasoactive agents such as NO, or to generate endothelium-derived hyperpolarization factors (EDHFs) to induce vasodilation, is a common underlining characteristic of a multitude of vascular pathologies [80,81]. Moraes et al. demonstrated here that following even only an acute period of creatine supplementation, microvascular reactivity and recruitment significantly improved, indicating improvements in EC function and possible increases in upstream contribution. Not to forget as well, creatine demonstrated the potential to lower both LDL-C and total cholesterol, both of which positively impact vascular health and reduce CVD risk [82]. Although no direct mechanisms by which creatine may have imparted these vascular benefits was assessed or reported, these findings still remain promising and bolster the need for further investigation into new novel mechanisms by which creatine may operate.

Van Bavel et al. [56] further studied the effect of creatine supplementation on the microvasculature, but chose to only include participants that followed a strict vegan diet that was rich in fruits and vegetables and void of any animal-derived foods. Considering that creatine is naturally found in meats and fish, it has been shown that those following a vegetarian or vegan diet present with substantially lower creatine stores than those ingesting an omnivorous diet [83]. Therefore, there is speculation that vegetarians or vegans may benefit from creatine to a greater extent, due to the potential to store more creatine during supplementation [83]. For this single-blinded, randomized study by Van Bavel et al., forty-nine vegan subjects aged between 20 and 45 years were separated into one of two major groups: creatine group (5 g/day for three weeks) or placebo group (5 g/day maltodextrin for three weeks). Similar to Moraes et al., blood specimens were collected before and after supplementation to determine any alterations in blood lipids (total cholesterol, LDL-C and HDL-C) and homocysteine. To determine the impact of supplementation on the microvasculature, laser speckle imaging with acetylcholine (ACh) skin iontophoresis was used to assess cutaneous microvascular reactivity, and intravital video-microscopy was used to measure skin capillary density and reactivity at both rest and following post-occlusive reactive hyperemia. The authors reported a reduction in homocysteine following creatine, however this was only reported in those previously presenting with elevated levels of homocysteine (hyperhomocysteinemia). Although regarded as statistically insignificant, there was also a trend towards the reduction in LDL-C and total cholesterol in those who ingested the creatine supplementation. In regard to the microvasculature, the authors reported that the basal capillary density of the creatine group was significantly increased in comparison to the placebo group. Furthermore, the authors reported a significant increase in capillary recruitment following post-occlusive reactive hyperemia for those in the creatine group but not those in the placebo group. These findings further support those reported by Moraes. Despite these promising results, the authors did not assess specific mechanisms by which creatine may have yielded these microvascular benefits. However, it was proposed in the concluding remarks that reductions in vascular oxidative stress (reduction in ROS) may have resulted in these vascular benefits; however, no biomarkers of oxidative stress were measured. Despite the lack of knowledge regarding creatine’s mechanistic contribution to vascular improvements, these findings further support the proposition that creatine may possess vascular-protective properties. 

The four above-mentioned clinical trials are the only studies to directly explore the effect of creatine on the vasculature, utilizing laboratory methods that are vascular-specific, such as PWV, venous occlusion plethysmography, laser speckle imaging and microscopy. Despite the scarcity of studies, Sanchez-Gonzalez et al., Moraes et al., and Van Bavel et al. all reported significant improvements in vascular variables following creatine supplementation regimens that ranged from 7–21 days, during which time participants consumed 100–210 g of creatine in total over the entire 7–21 days. Furthermore, even though benefits were not reported by Arciero et al. following creatine supplementation alone, there was a noticeable and significant synergistic improvement when this supplement was combined with resistance training. It is also important to highlight the primary commonality, and possible limitations, shared between these studies, in that all studies utilized relatively young, healthy individuals as study subjects. Although the development of vascular pathology and CVDs are multifactorial and can be influenced by a variety of lifestyle factors, the primary unmodifiable risk factor for CVD is age [84,85], hence the vast proportion of CVD deaths occurring in those aged 75 years and above [2]. Therefore, when considering the integrity of the cardiovascular system of these young, healthy individuals, it seems justified to assume that no vascular dysfunction, or need for improvement, was even present prior to study involvement. Thus, if these authors reported benefits to the vasculature, albeit minor but statistically significant, in this young demographic, one could argue that the benefits of creatine may be even greater when applied in a population already at risk of vascular dysfunction (chronic smokers, elderly, post-menopausal women), or those suffering from CVDs already. Hence, there is a clear need for further, more population-specific, investigation into the potential of creatine to benefit vascular health.

## 4. The Presence and Function of Creatine within the Vascular Endothelium

Prior to proposing potential mechanisms by which creatine may be able to serve as a therapeutic supplement for vascular health, it is important to note the presence of creatine and its functional constituents within the vascular endothelium specifically. 

Creatine that has either been consumed naturally via the diet or through supplementation is actively absorbed across cell membranes into the intracellular compartments via a creatine transporter (CRT), also known as SLC6A8. This transporter is sodium-(Na^+^) and chloride-(Cl^−^) dependent, requiring at least two Na^+^ ions and one Cl^−^ ion for the transport of one creatine molecule [86]. Although the vast majority of creatine stores can be found within human skeletal muscle (~95%) [49], the presence of the CRT has been identified on enterocytes [87], kidney epithelial cells [88], the blood–brain barrier [89], and the ECs that line the vascular system [90]. Once located intracellularly, creatine can exist either in a free form or in a phosphorylated form, phosphocreatine (PCr). Both creatine and PCr, together with globally present creatine kinase (CK) isoenzymes, function as high-energy compounds crucial for cellular metabolism, working as the known creatine-phosphocreatine system (Cr-PCr system) [9]. In short, during cases of low cellular adenosine triphosphate (ATP) levels or high energy demand, CK will catalyze the transfer of the *N*-phosphoryl group from PCr to adenosine diphosphate (ADP) to resynthesize ATP and replenish the cells intracellular ATP pool. Conversely, when ATP production from either glycolytic or oxidative pathways are greater than ATP utilization, CK can function in reverse to capture and store this cellular energy by replenishing PCr stores. For further, more in-depth information regarding the cellular intricacies of the creatine system, readers are encouraged to read reviews by Wallimann et al. [91] and Persky and Brazeau [92]. In addition to its role as a temporal high-energy phosphate buffer, considering the complexity and variety of CK isoenzymes and their subcellular compartmentalization and distribution, the Cr-PCr system is also believed to function as a spatial high-energy shuttle. This shuttle serves to quickly, and efficiently, shuttle high-energy phosphates (potential energy) between sites of ATP production (such as the mitochondrial electron transport chain) and sites of ATP utilization (such as ATP-gated ion channels, ATP-regulated receptors, ATP-regulated ion pumps; contractile processes, cell motility, cell signaling, or organelle transport [93]). Taking into consideration the complexity and vast functionality of the Cr-PCr system, it is clear that creatine plays a vital role in cellular function. 

Decking et al. [90] investigated the importance and functional aspects of CK isoenzymes in a variety of ECs throughout the vascular system. Using both the ECs of the aorta (AECs) and the microvasculature (MVECs) of pigs and rats, Decking looked to assess the presence of varying CK isoenzymes in each subtype of ECs, the intracellular concentration of energy phosphates, and the function of CK isoenzymes during substrate depletion. First and foremost, supporting that of other studies [94,95], Decking reported that each variety of EC demonstrated the ability to take up creatine from the medium, thus indicating the presence and function of an EC-CRT. Through the use of phosphorus-31 nuclear magnetic resonance (31^P^-NMR) spectroscopy to monitor cellular energy status, Decking also illustrated that porcine AECs contained a considerable amount of PCr, which suggested the presence of cytosolic CKs. Furthermore, when incubated in a creatine-rich medium, AEC concentrations of PCr increased, thus confirming the presence of a CRT and Cr-PCr system. Interestingly, when the medium was devoid of creatine, PCr accumulation rate was reduced by >90%. Decking had ultimately demonstrated not only the presence of PCr in both types of ECs, but that these concentrations were reversible, thereby indicating intricate intracellular control of energy substrates via a CK pathway. In regard to specific isoenzymes, Decking reported only the brain-brain CK (BB-CK) isoenzyme being present in the cytosol of cultured macro- and micro-ECs. However, both rat and pig ECs presented with mitochondria-specific CKs (Mt-CKs). Taking into consideration the finding of both a cytosolic CK and mitochondrial CK, Decking concluded that this strongly suggested the presence of an active Cr-PCr system, or energy shuttle between compartments. Although the expression and activity of CK and the Cr-PCr system varies between vascular beds, this pioneering research supports the presence and function of creatine and the Cr-PCr system within the vasculature. Other notable studies that complement the findings of Decking et al., supporting the presence and function of creatine, CRT and CK in ECs, include those by Loike et al. [96], Nomura et al. [54], and Sestili et al. [97]. 

## 5. Possible Application of Creatine for the Promotion of Vascular Health

Taking into consideration the additional non-energy related properties of creatine, and the beneficial application of this supplement within other pathologies [98,99], there is evidence to suggest that creatine may contribute to other novel mechanisms which could therapeutically benefit vascular health. However, scientific evidence of potential novel mechanisms is sparse. The following sections provide a unique perspective on creatine, highlighting some promising properties of creatine that may contribute to vascular health in a novel way, such as the impact of creatine on oxidative stress, nitric oxide (NO) and endothelial nitric oxide synthase (eNOS), endothelium-derived hyperpolarization factors (EDHFs), endothelial cell integrity, and the protection of cellular deoxyribonucleic acid (DNA) and ribonucleic acid (RNA).

### 5.1. Creatine, Oxidative Stress and Nitric Oxide Bioavailability 

Oxidative stress is a pathological state of imbalance between the production of damaging free radicals and their removal by antioxidant defenses [100]. The highly reactive, unstable free radicals that characterize oxidative stress can be formed from many compounds including oxygen and nitrogen, forming reactive oxygen species (ROS) and reactive nitrogen species (RNS), respectively; or, reactive oxygen and nitrogen species (RONS) collectively [101]. Unsurprisingly, the presence or progressive accumulation of oxidative stress has been found to underline a multitude of diseases, including CVDs such as hypertension, atherosclerosis, and stroke [67]. Furthermore, other notable lifestyle risk factors that are closely associated with CVD such as smoking, alcohol abuse, lack of physical activity, and poor diet (high fat intake) [10], similarly contribute to the production of harmful free radicals [102]. Free radicals, being inherently unstable, often steal electrons from other biomolecules to satisfy their need for valent shell completion. As a consequence of this, lipids, proteins, carbohydrates, RNA and even DNA can become oxidized (loss of an electron), which alters their structure and often renders them dysfunctional. Fortunately, biologically we possess an intricate antioxidant system poised to protect, quench and to reduce the accumulation of free radicals before they can impose their harmful effects. The antioxidant system is diverse in nature, expressing both enzymatic and non-enzymatic processes which function to either directly or in indirectly reduce free radicals. However, despite these natural defenses, the antioxidant system has been shown to slowly diminish and become overwhelmed as a result of biological aging [103], in addition to being significantly impaired by certain factors such as smoking [104], poor nutrition [105] and lack of physical activity [106]. Despite the lifelong pressure put upon our antioxidant defenses, fortunately antioxidants also exist in a variety of natural sources that we can ingest through our diet. For example, foods rich in polyphenols [107], carotenoids [108], lycopene [109], vitamins (A, C and E) [110], and flavonoids [111], have all been shown to support the body’s natural antioxidant defenses. It is due to the benefit of these naturally occurring antioxidants that antioxidant-containing foods and supplements have been well researched [44,112,113] and used as a therapeutic strategy for the treatment of many diseases. It is important to note that antioxidant supplementation, as with medications and other therapies, is not without limitations. Despite promising in vitro data and the emphasis put upon positive clinical trials, other clinical research has produced few promising results, reporting a variation in effectiveness between diseases and populations, with further issues surrounding individual antioxidant bioavailability and metabolism [114,115]. Therefore, although supplemental antioxidant protection is theoretically sound and physiological promising, further clinical research is still required to expand this field. 

Considering the above findings, the question that remains is where does creatine supplementation fit into the realm of antioxidant defenses against oxidative stress, and ultimately improvement in vascular health? Despite creatine being structurally different from other natural antioxidants, and rarely being marketed as a potential antioxidant, there is evidence to suggest that creatine may possess both in-direct and direct-antioxidant properties [9,52,53]. For an in-depth perspective on creatine and its antioxidant potential readers are suggested to read that by Sestili et al. [53]. Matthews et al. [62] was one of the first to investigate the potential of creatine to protect against oxidative stress and neurotoxicity induced by intrastriatal injections of malonate or intraperitoneal injections of nitropropionic acid (3-NP), an animal model of Huntington’s disease. It was first reported that following two weeks of 1% dietary creatine supplementation, PCr concentrations within striatal slices significantly increased, suggesting the presence of a CRT and the ability of creatine supplementation to increase brain stores of energy metabolites. Furthermore, the authors reported that those animals consuming creatine for just two weeks had significantly reduced striatal lesions following both malonate and 3-NP neurotoxicity, suggesting significant neuroprotection. Finally, Matthews reported that creatine reduced markers of hydroxyl (·OH) free radical generation. These findings led to the first postulation that creatine may infer antioxidant-like properties. Following this study, Lawler et al. [52] investigated the direct antioxidant properties of creatine. Lawler, using a highly controlled acellular setting, looked to determine the impact of varying doses of creatine on five ROS systems: xanthine oxidase for superoxide anions (O_2_^−^), hydrogen peroxide (H_2_O_2_), peroxynitrite (ONOO^−^), lipid peroxidation, removal of 2,2′-azino-bis3-ethylbenzothiazoline-6-sulphonic acid (ABTS^+^) cation radical, and tert-butyl-hydroperoxide (tBOOH). Antioxidant scavenging capacity (ASC) was also assessed. Following these experiments, authors reported that creatine exhibited significant oxidant scavenging potential of ionized radicals such as ABTS^+^, O_2_^−^, and ONOO^–^. Furthermore, there was a direct dose–response relationship found between creatine and total ASC. All in all, despite being in a controlled acellular setting, Lawler was the first to show the direct antioxidant potential of creatine, thereby sparking the need and interest of further research both in vivo and in vitro. To approach the issue regarding the need for further in vitro data, Sestili et al. [97] investigated the ability of creatine to serve as an antioxidant within animal (murine myoblasts–C2C12) and human (promonocytic–U937; umbilical vein endothelial cells–HUVECs) cultured cell lines that were oxidatively injured with H_2_O_2_, tBOOH, and ONOO^−^. Sestili reported that creatine treatment significantly reduced the cytotoxic effects of all oxidative stressors, consequently increasing cell vitality. Supporting that previously found by Lawler, Sestili also reported a significant dose-dependent effect. Although no exact mechanism was offered by Sestili, it is interesting to note that cellular concentrations of creatine increased following supplementation, but had no impact upon antioxidant enzymes. Furthermore, the antioxidant effects of creatine were abolished following the addition of a CRT inhibitor, β-guanidinopropionic acid. This excitingly suggests that creatine offered these antioxidant effects directly and were dependent upon intracellular creatine concentrations. In addition to the promising findings outlined above, other studies that similarly support the ability of creatine to protect and serve as an antioxidant include that by Fimognari et al. [116], Rambo et al. [117], Sestili et al. [118], and Hosamani et al. [119]. 

Although the above aforementioned studies all elude to the ability of creatine to serve as an antioxidant, most studies utilized oxidative stressors that were externally derived (i.e., controlled in the experiment, added to the medium, added to the cell lines). Meyer et al. [58] however, looked to determine whether creatine could directly serve as an antioxidant against one of the largest contributing sources of ROS in the body, the mitochondria. Mitochondria are complex organelles whose primary function is respiration, or oxidative phosphorylation, promoting energy production in the form of ATP through a series of intermittent stages termed the electron transport chain (ETC). Both O_2_^−^ and H_2_O_2_ are produced following the leakage of electrons from varying redox centers of the ETC and other associated metabolic enzymes. Eleven distinct mitochondrial sites of O_2_^−^ and/or H_2_O_2_ production have been identified, each with unique properties and contributing to mitochondrial ROS (mtROS) formation in varying amounts [120]. Although, in small amounts, mtROS is necessary for certain physiological processes such as cell signaling [120], high production or accumulation of mtROS has been closely associated with cell death (apoptosis) and cellular energy dysfunction, and has been shown to be associated with a multitude of vascular pathologies such as atherosclerosis, stroke, and hypertension [67]. The rate of mtROS production has been found to be dependent upon mitochondrial membrane potential, cellular PCr/Cr ratios, and adenine concentrations [121]. Adequate creatine availability and functional mitochondrial creatine kinases (mt-CK) have similarly been found to be necessary for the maintenance of ADP/ATP ratios that are favorable for the respiratory chain to proceed at sustainable, low ROS producing rates [58]. Meyer et al. was the first to show that exogenous creatine supplementation was capable of stimulating efficient mt-CK function, through the augmentation of cellular PCr/Cr ratios and stabilization of cellular ADP/ATP ratios. These collectively led to a reduction in H_2_O_2_ production through mt-CK-dependent ADP recycling in adult male Wistar rats. An additional study by Barbieri et al. [122] also demonstrated the protective effect of creatine supplementation upon the mitochondrial membrane potential, thereby sustaining the efficiency and integrity of the mitochondria in oxidatively injured (H_2_O_2_ treated) C2C12 mouse myoblasts. All in all, these findings support the ability of creatine to not only serve as an antioxidant, but to also successfully reduce ROS production by facilitating healthy mitochondrial function, which could therefore help reduce the risk of developing vascular pathologies

Despite the growing body of knowledge around creatine as an antioxidant, very few studies have looked at the ability of creatine to exhibit these same properties in humans. Rahimi et al. [123] investigated the effect of creatine supplementation (4 × 5 g/day for 7 days) on exercise-induced oxidative stress following an acute bout of resistance exercise. This double-blind, placebo-controlled study utilized twenty-seven healthy young men, and assessed oxidative stress through plasma malondialdehyde (MDA) and urinary 8-hydroxy-2-deoxyguanosine (8-OHdG) immediately and 24-h post exercise. Rahimi reported that those supplemented with creatine presented with significantly lower markers of 8-OHdG immediately and 24-h post exercise, in comparison to placebo. Furthermore, plasma MDA concentrations were significantly higher in the placebo group post exercise. Rahimi concluded that these results supported the ability of creatine to reduce oxidative damage induced by acute resistance exercise. Despite these promising findings, however, further research is still needed to fully determine the application of creatine as an antioxidant in humans specifically. 

Taking into consideration that many, if not all, CVDs are characterized by oxidative stress and the subsequent vascular dysfunction [67,113,124,125,126], the ability of supplemental creatine to potentially serve as an antioxidant is just one of the novel ways in which it may be able to benefit vascular health. Further expanding upon the promising implication that creatine may serve against oxidative stress, it could be proposed that creatine could benefit vascular health through its impact on nitric oxide (NO) and endothelial nitric oxide synthase (eNOS). 

One of the primary functions of healthy vascular ECs is to synthesize and release vasoactive factors to aid in the control of vasomotor tone, stimulating vasodilation or vasoconstriction to sustain healthy blood flow and regulation. NO, first described by Furchgott & Zawadzki in 1980 [127], is one of the most crucial endothelium-dependent vasodilators in the vascular system [128]. NO is synthesized in vascular ECs by the specific eNOS enzyme, in which l-arginine is converted into NO and citrulline [129]. NO exerts its vasodilatory properties in a paracrine manner by diffusing from ECs into adjacent VSMCs. The inhibition of NO via the perfusion of NG monomethyl-l-arginine (L-NMMA) has been shown to result in a dose-dependent increase in blood pressure due to increased global vasoconstriction, which can then be reversed by reintroducing NO [130]. Furthermore, even though NO is technically a free radical by definition, there is a clear association between NO-deficiency and CVD risk; with a decrease in NO bioavailability or eNOS dysfunction being linked to atherosclerosis [131], hypertension [132], Type II diabetes [133], arterial stiffness [134], stroke [135], heart disease [80], and overall mortality [136]. Clearly, sufficient NO is necessary for vascular health. Unfortunately, due to the short half-life and diffusion distance of around 1–10 s and 50–1000 μm, respectively, NO is often targeted and rendered biologically futile by the presence of other ROS such as O_2_^−^ [137]. In fact, the rate at which NO and O_2_^−^ react occurs at the near-diffusion-limited rate of 6.7 × 109 M^−1^·s^−1^ [138]; therefore, nearly every collision between O_2_^−^ and NO results in the irreversible formation of ONOO^−^, another biologically relevant cytotoxic free radical [139]. Thus, in situations in which oxidative stress is high, overwhelming the antioxidant system, the bioavailability of NO can be detrimentally reduced, thereby impairing vascular function. Evidence of this can be seen in populations characterized by heightened levels of oxidative stress, in which NO bioavailability is low and vascular health and function is impaired, such as the elderly [140] and those with pathologies such as chronic kidney disease [141,142]. In addition to this, oxidative stress has also been shown to target important co-factors necessary for eNOS to efficiently synthesize NO. For example, free radicals have been shown to oxidize tetrahydrobiopterin (BH_4_) into dihydrobiopterin (BH_2_), consequentially leading to eNOS uncoupling, the reduction in NO production, and the potentiating of preexisting oxidative stress via the production of RNS [143,144]. The uncoupling and oxidative damage to eNOS has also been linked closely with a variety of CVDs [144,145,146]. 

Taking into consideration that oxidative stress can hinder NO’s synthesis, function and bioavailability, if creatine exhibits antioxidant properties it could be hypothesized that creatine may mechanistically aid in the reduction and scavenging of ROS, thereby increasing NO bioavailability and contributing to improved vascular health. Ahsan et al. [147] investigated this novel theory and assessed the role of PCr in HUVECs injured by oxidized low-density lipoproteins (OxLDLs), a consequence of ROS, and its influence on the eNOS pathway. Ahsan reported that following damaged induced by OxLDL insult, HUVECs pre-treated with 10–30 mM of PCr had significantly reduced signs of endothelial apoptosis (cell death), reduced ROS generation and improved NO content. Interestingly, Ahsan also reported that those HUVECs treated with PCr had sustained eNOS signaling via the phosphatidylinositol 3-kinase/protein kinase B/endothelial nitric oxide synthase (PI3K/Akt/eNOS) pathway. Ahsan therefore hypothesized that these PCr-mediated benefits were due to, in part, the antioxidant activity of PCr and the ability of PCr supplementation to modulate the PI3K/Akt/eNOS pathway. In addition to this, there is further evidence that demonstrates the ability of other supplemental antioxidants to improve NO bioavailability and eNOS coupling through ROS reduction, thereby supporting vascular health [148,149,150,151,152,153]. Taking all of this into consideration, if creatine can truly serve as an antioxidant and potential eNOS-stimulating supplement, creatine could serve as a vascular-protective supplement. However, more clinical trials are needed to quantify and to truly measure whether creatine supplementation could provide vascular benefits through this novel antioxidant, eNOS stimulating, NO increasing mechanism, especially in humans.

### 5.2. Creatine and Endothelium-Derived Hyperpolarization Factors

In addition to the important endothelium-derived relaxing factor NO, the endothelium is known to stimulate two additional relaxing factors, prostacyclin (PGI_2_—via the cyclooxygenase pathway [154]) and endothelium-derived hyperpolarization factor (EDHF) [155]. Although the contribution of NO and PGI_2_ to endothelium-dependent vasodilation and control of both local and systemic blood pressures is well established [156], the exact contribution of EDHFs are less known. Nevertheless, evidence suggests that EDHFs play a vital role in the control of vasomotor tone, working in synergy with other vasoactive factors to similarly contribute to blood pressure regulation [157]. For example, Scotland et al. reported that following the inhibition of both NO and PGI_2_ pathways, vasodilation still occurred following stimulation, highlighting the key compensatory role of EDHFs [158]. Furthermore, it has been shown that a reduction in the potential of ECs to stimulate EDHFs is closely associated with increased CVD risk [159,160,161,162]. 

As made apparent by its given name, EDHFs stimulate vessel vasodilation through the hyperpolarization of neighboring VSMCs. The hyperpolarization initialized within ECs can either be propagated to neighboring VSMCs directly, via specialized myo-endothelial gap junctions, or trigger an increase of potassium (K^+^) ion efflux into the subendothelial space. The resulting increase in K^+^ within the interstitium can then activate calcium-dependent potassium channels (KCa^+^), inwardly rectifying potassium channels (KIR^+^), or the sodium-potassium pump (Na^+^-K^+^ pump) on VSMCs, causing VSMC hyperpolarization. This hyperpolarization then causes the closure of smooth muscle voltage gated calcium (Ca^2+^) channels, resulting in the reduction of intracellular Ca^2+^ concentrations and ultimately vessel relaxation [155]. The initial EC derived hyperpolarization can be stimulated by a variety of stimuli, including acetylcholine (ACh), mechanical factors such as shear stress, and bradykinin. Upon stimulation by varying agonists, intracellular levels of Ca^2+^ within the ECs increase, thereby activating endothelial-specific KCa^+^ channels that initialize the original hyperpolarization. Currently, both myo-endothelial gap junction coupling and fluctuations in K^+^ specifically (intracellularly and intercellularly), remain comparably important and appropriate explanations for EDHF. However, it is clear by the vast involvement of multiple channels, pumps and specialized gap junctions, that the mechanism by which EDHFs function is multifactorial. It is evidential that the pathological disruption or dysfunction of any of these structures would lead to the impairment of EDHF stimulation, thereby reducing healthy vascular function. 

Among the variety of influential K^+^ pumps that contribute to EDHFs and vascular tone regulation, is the ATP-sensitive potassium pump (KATP). The KATP has been found to be widely distributed in a variety of tissues, including cardiac tissue (cardiac myocytes [163]), pancreatic β cells [164], vascular ECs and VSMCs [165,166,167,168]. The KATP channel consists of four pore-forming inward rectifier K^+^ channel subunits, in addition to four sulfonylurea receptors (SUR) [168]. This specific KATP channel has been demonstrated to play a crucial role in vascular tone regulation in a variety of studies utilizing pharmacological approaches [169,170], transgenic mouse models [171,172], and in human patients [173,174]. KATP channels, hence their name, are subjected to regulation by intracellular ATP and ADP levels. High amounts of intracellular ATP reduces the vascular KATP channel activity (closure), whereas ADP concentrations between 0.1–3 mmol/L causes stimulatory (opening) effects [175]. The inhibition, or blockage, of KATP channels has been shown to decrease the extent of vasodilation in cerebral arterioles, basil arteries, coronary arteries, mesenteric arteries and internal mammary arteries [168]. Taking into consideration the evidence that supports the role in which KATP channels play in vascular tone, any dysfunction or deterioration of these channels is likely to contribute to the pathogenesis of many CVDs. 

Therefore, how may creatine play a potential role in the support of EDHFs, thereby supporting and potentially improving vascular health and function? As stated previously, the Cr-PCr system functions as both a high-energy phosphate buffer and a spatial high-energy phosphate shuttle [93]. Considering that KATP channels are regulated by ADP and ATP, and that the Cr-PCr-system helps regulate metabolites within the cytosol and locally around ATP-consuming proteins [93]; one could claim that through creatine supplementation, KATP channels could be benefitted. In a well-written review by Dzeja & Terzic [176], the authors highlighted how the microenvironment of these specific channels harbors several phosphotransfer enzymes, including CKs, that enable the local transfer and control of ADP and ATP. It was concluded that through the delivery and removal of adenine nucleotides at these KATP channels, that the ATP/ADP ratio could be controlled, thereby influencing the opening or closing of the channel and vascular tone regulation. Other studies, such as that by Selivanov et al. [177], further support the presence of CK enzymes coupled to these specific KATP channels. Selivanov suggested that it is the collaboration between CKs and adenylate kinases (AKs) that serves as the metabolic sensor of KATP channels, in addition to controlling intracellular phosphotransfer fluxes, the regulation of channel function and vascular response. In other words, increased intracellular levels of creatine in tissues such as the endothelium, could help regulate KATP channels, hyperpolarizing the neighboring VSMCs, and contributing to the enhancement of EDHF mediated vasodilation and ultimately vascular health. It is this hypothesis that Moraes et al. claimed could have led to the improvement in microvascular reactivity and density he reported following creatine supplementation [55]. 

Another study that could potentially support the hypothesis of creatine supporting the function of EDHFs, is that conducted by Guerrero and colleagues [178]. Guerrero et al. looked to investigate the impact of varying ATP-generating systems upon the function of the Na^+^-K^+^ pump, also known as the Na^+^-K^+^-ATPase. As noted previously, the Na^+^-K^+^ pump also contributes to the generation of VSMC hyperpolarization, and quite like the described KATP channel, is ATP-dependent. Following the inducing and inhibition of varying ATP-generating systems (glycolysis, oxidative phosphorylation, and the CK-system), Guerrero concluded that even in the absence of glycolytic and oxidative ATP, the CK system and 3 mM of supplemental PCr was able to support the ATP supply required for Na^+^-K^+^ pump activity. Although this study was conducted in kidney cell epithelia, these results suggest a role of the CK system and supplemental PCr in the function of the Na^+^-K^+^ pump; a pump that has been evidentially shown to also contribute greatly to the stimulation of EDHFs. Therefore, this could justify the need for similar experiments investigating the role of creatine and ATP-dependent pumps in vascular cells specifically. 

Despite the lack of direct evidence surrounding the impact or contribution of creatine supplementation towards the function of pumps, channels and structural junctions involved in the development of EDHFs, the evidence provided here outlines another novel mechanism by which creatine could potentially benefit vascular health. This evidence further emphasizes the need for future clinical trials, both in vivo and in vitro, to identify the role creatine and the Cr-PCr system in EDHFs. 

### 5.3. Creatine, Endothelial Cell Integrity, and Inflammation 

As stated in previous sections, the ability of ECs to synthesize and release a variety of vasoactive factors is paramount for overall vascular health [17], with the manifestation of ED substantially increasing the risk of CVD development [15]. In addition to ED however, endothelial structural integrity and permeability is also closely associated with EC health and CVD risk [179]. ECs line the entirety of the vascular system, directly facing the vessel lumen. Therefore, ECs serve as a semi-permeable barrier between the blood and its circulating components (water, nutrients, hormones, proteins) and the underlining tissues, allowing for the selective movement and diffusion of both small and large molecules [180]. Evidence has shown that any destabilization in the junctions that connect each EC, or disruption in the endothelial barrier itself can result in increased permeability (also known as “endothelial leakiness”) and an increased risk of CVDs such as atherosclerosis, coronary artery disease, stroke, and thrombosis [179,181]. 

Vascular EC barrier function has been shown to be critically supported by intercellular junctions located between neighboring ECs [182]. Two major subtypes of intercellular junctions include tight junctions (TJ) and adherens junctions (AJ); however, it is the TJs that are majorly responsible for barrier function and the control of EC permeability [183]. In healthy physiological conditions, vascular ECs are regulated, and permeability is intricately controlled. In vascular pathologies however, proinflammatory signals activate the expression of varying adhesion molecules and the attraction of damaging leukocytes that collectively destabilize and debilitate the endothelial barrier [179]. It is evident in diseased vessels that barrier function of the ECs has been weakened, resulting in increased permeability and pathological alterations in EC structure [179]. Risk factors that are associated with these pathological changes include dyslipidemia [184], diabetes [185], obesity [186], and smoking [187], all of which lead to chronic inflammation and the accumulation of ROS (oxidative stress), which are also risk factors to barrier dysfunction [179]. Considering the destructive consequences of such risk factors, therapeutically reducing these risk factors and increasing the stability of TJs and the EC plasma membrane, could collectively help prevent the manifestation of endothelial barrier dysfunction. 

Nomura et al. [54] was among the first to report on the impact of creatine on inflammation and EC cell membrane permeability. Using pulmonary ECs in culture, Nomura demonstrated that upon incubation with a creatine, EC intracellular stores of creatine and PCr significantly increased, signifying the presence of a CRT and CK system. When looking closer at the impact of this increased creatine and PCr, Nomura reported that following 5 mM of supplemental creatine, neutrophil adhesion to ECs had significantly reduced. Additionally, following only 0.5 mM of creatine, the expression of inflammatory markers such as intercellular adhesion molecule-1 (ICAM-1) and E-selectin was similarly reduced. Taking into consideration the known impact these inflammatory markers have upon endothelial permeability and CVD [188,189]; these results suggest potential benefits for creatine and EC health. Nomura’s reported benefits, however, did not stop at a reduction in inflammatory markers. Nomura also reported that following cytotoxicity induced by serotonin and H_2_O_2_, 5 mM of supplemental creatine significantly reduced EC permeability, improved EC membrane stability, and reduced overall EC leakiness. Collectively these findings support the hypothesis that creatine may benefit EC stability, vascular protection, and health.

Similarly investigating the role of creatine in EC stability, Tokarska-Schlattner et al. [190] proposed a novel mechanism in which creatine, due to its zwitterionic structure, may directly interact with membrane phospholipids. Schlattner directly tested the lipid interaction of both creatine and PCr, in addition to the cyclic analogues cyclo-creatine (cCr) and phospho-cyclo creatine (PcCr), using liposome model systems. Following surface plasmon resonance spectroscopy, authors reported a low affinity PCr/phospholipid interaction which additionally induced changes in liposome shape, indicating alterations in the lipid bilayers. In addition to this, authors reported that PCr efficiently protected against induced membrane permeabilization in two separate models: induced liposome-permeabilization by membrane-active peptide melittin, and erythrocyte hemolysis induced by doxorubicin (oxidative drug), hypoosmotic stress or saponin (mild detergent). These excitingly novel results were the first to demonstrate the potential of guanidino compounds, such as PCr, to interact directly with membrane phospholipids, resulting in the modulation of membrane properties and membrane stabilization. The authors thereby concluded that PCr, which could be augmented by supplemental creatine, could exert protective effects upon cell membranes under pathological conditions. Therefore, creatine supplementation may be able to stabilize EC membranes, attenuate EC leakiness, increase EC health and protect against vascular pathologies. Furthermore, taking into consideration the presence of varying cell signaling molecules embedded within the lipid bilayer, such as G-protein coupled receptors, mechanotransducers and bioactive lipids, it could be speculated that the insertion of PCr within the lipid bilayer could have stimulatory or inhibitory effects on these molecules, thereon impacting downstream pathways and protein signaling. This speculative proposal, however, requires further investigation.

As previously mentioned, varying disorders such as diabetes have been shown to both lead to and be characterized by endothelial leakiness. Considering the pleiotropic benefits of creatine, Rahmani et al. [185] looked to determine the effect of creatine supplementation on serum biochemical markers associated with diabetes [triglycerides (TGs), total cholesterol, LDL-C, and HDL-C], and the endothelial permeability of coronary arteries within diabetic rats. Thirty-two Wistar rats were allocated randomly into one of four groups: control; supplemented creatine; diabetic; diabetic and creatine. Supplementary creatine monohydrate was given to those allocated at a dose of 400 mg/kg/daily for five months. Rahmani reported that following the supplementation period, those rats undergoing creatine treatment had significantly reduced serum levels of TGs, cholesterol, and LDL-C, with an additional positive increase in the more vascular-protective HDL-C. Furthermore, coronary permeability was significantly reduced in the diabetic group treated with creatine supplementation, in comparison to other un-treated groups. The authors therefore concluded that not only did creatine serve as a lipid-lowering supplement but, similar to that shown by Nomura et al. and Schlattner et al., creatine also reduced EC permeability and leakiness.

Recently, Hall et al. [191] identified a unique requirement for creatine and the Cr/PCr system within intestinal epithelial cells (IECs). Those suffering from irritable bowel disease (IBD) have been shown to suffer from intestinal barrier dysfunction, or intestinal epithelial leakiness. In a previous animal model of colitis, evidence suggests that creatine regulates energy distribution within IECs and further reduces the severity of colitis [192]. Hall and colleagues therefore looked to determine the importance of the CRT and Cr/PCr system within human patients suffering from IBD, and in an animal model in which mice presented with or without the CRT gene. Interestingly it was reported that the CRT was found localized around the TJs of IECs, and that the CRT closely regulated the intracellular creatine concentration and resultant barrier formation. Furthermore, in the absence of creatine, the stability of TJs was significantly altered, resulting in increased intestinal leakiness. Finally, in human biopsies, those suffering from IBD had reduced levels of messenger-RNA encoding for the CRT. It was concluded by the authors that the CRT regulates the energy balance in IECs needed for the sufficient function of TJs; therefore, it is the CRT and intracellular creatine that regulates epithelial integrity and barrier function. Despite this study being conducted in IECs specifically, what is interesting here is that Hall et al. provides evidence that illustrates the need of operational CRTs and sufficient intracellular creatine to maintain the efficient function of TJs. As alluded to earlier, in ECs specifically, the healthy function of TJs is paramount for EC integrity and barrier health. Therefore, one could hypothesize that through creatine supplementation and an increase in EC intracellular creatine concentrations, TJ energy requirements could be sufficiently managed, leading to efficient function of TJs, improved integrity and reduced risk of EC leakiness. 

EC leakiness underlines a multitude of CVDs and can be augmented by a variety of risk factors such as inflammation, oxidative stress, diabetes and dyslipidemia. Thus far, we have already outlined the potential for creatine to act as an antioxidant, which in itself could have positive benefits upon vascular EC membrane stability. In addition to this however, there is evidence to suggest that creatine supplementation could reduce circulating lipids, reduce EC leakiness caused by inflammation and diabetes, and aid in the maintenance of TJ function. Despite the novelty of these properties, and the lack of true clinical trials, again we are left with what could be another benefit of creatine supplementation, one that could benefit vascular health through the improvement in EC integrity and reduction in EC leakiness.

### 5.4. Creatine and Vascular DNA/RNA Protection

Without the protection, maintenance, and accurate repair of DNA in response to disruption or damage, the intricate process of DNA replication and genetic coding can fail. Conclusively, the integrity of DNA is vital for cellular health, and unless precisely repaired in the event of corruption, nuclear DNA and mtDNA damage can lead to devastating mutations or other deleterious cellular consequences [193]. Damage to both DNA and ribonucleic acid (RNA) has been found to underline a plethora of vascular pathologies such as hypertension [194], coronary artery disease [195], atherosclerosis [196], and peripheral artery disease [197]. Furthermore, damage to mtDNA specifically has been closely associated with a decline in mitochondrial function, increase in mtROS production, mitochondrial apoptosis, and the manifestation of CVDs [198]. Although DNA and RNA damage can be a natural consequence of the aging [193,199], damage can also be elicited by factors such as ROS [200], radiation (such as ultraviolet rays [201]), and chemotherapeutic drugs [202]. Taking into consideration that lifestyle factors such as obesity [203], poor nutrition [204], smoking [29], and alcohol abuse [205] all lead to an increase in ROS, one could connect these lifestyle factors to an increased risk of DNA and RNA damage also. Despite DNA being a complex target for therapeutics, some promise has been found within the use of RNA therapeutics for the treatment of CVDs [206] and vascular pathologies specifically [207,208]. However, these therapeutic strategies are still relatively new; therefore, reducing inducers of DNA and RNA damage, such as ROS, could serve as a more realistic target for immediate treatment strategies. 

In addition to the plethora of non-energy related properties exhibited by creatine supplementation, creatine has also been shown to protect against a variety of DNA and RNA damage. Berneburg et al. [209] investigated the impact of creatine on mitochondrial mutagenesis, function and mtDNA damage in cultured human skin fibroblasts, exposed to intense UV-radiation. Following UV-radiation, mtDNA was extracted and analyzed using real-time polymerase chain reaction (PCR) to determine DNA damage, and complete mitochondria were analyzed for oxygen consumption, ATP content, and mitochondrial membrane potential, used as markers for mitochondrial function. Following UV exposure, mtDNA was significantly damaged, resulting in a decrease in mitochondrial function. The authors further reported however, that mtDNA damage and mutagenesis, in addition to functional assessments, were all normalized and improved by increasing intracellular creatine levels. Furthermore, quite like that of other studies, creatine supplementation resulted in a dose-dependent increase in intracellular creatine concentration. Berneburg concluded here that increases in intracellular creatine played a significant protective role, protecting fibroblasts from functional-deteriorations and mtDNA damage induced by radiation. 

Barbieri et al. [122] further investigated the impact of creatine supplementation on mitochondrial biosynthesis and mtDNA in differentiating C2C12 cells under oxidative conditions. Prior to analysis, creatine was preloaded to C2C12 cells by adding either 3 mM or 10 mM over the first 24 h of differentiation. Cultures were then exposed to 0 or 0.3 mM H_2_O_2_ for 1-h to induced oxidative stress. In regard to cell vitality, authors reported that creatine supplementation of just 3 mM significantly improved cell vitality in response to oxidative challenge. Barbieri further reported that creatine supplementation significantly increased mitochondrial markers of biosynthesis, and aided in the protection of mtDNA. Barbieri concluded that these results further supported the notion that creatine was capable of protecting mitochondria and mtDNA against oxidative insult. Considering the association between mtDNA damage, mitochondrial apoptosis and vascular pathologies [210,211,212], these findings suggest a potential benefit of creatine to support vascular health. On another interesting note, Barbieri found that creatine was capable of activating adenosine monophosphate-activated kinase (AMPK) within C2C12 cells. In ECs specifically, AMPK can phosphorylate and stimulate eNOS, thus the synthesis of NO. Therefore, this could be another unique and novel molecular pathway to investigate in future studies regarding creatine and vascular health. 

In addition to those noted above, other studies that support the role of creatine supplementation in the protection of DNA damage include that by Rahimi et al. [123] and Mirzaei et al. [213], who both report the ability of creatine to protect DNA against oxidative stress induced by exercise. Furthermore, Qasim & Mahmood [214] report findings that suggest a DNA-protective role of creatine in human erythrocytes and lymphocytes oxidatively challenged by H_2_O_2_. 

In an attempt to explore creatine’s role in DNA protection within the vasculature specifically, Guidi et al. [215] looked to evaluate the protective effects of creatine supplementation on oxidatively injured nuclear DNA (nDNA) and mtDNA, in both an acellular system using plasmid DNA and in vascular specific cultured HUVECs. To induce oxidative damage, HUVECs pretreated with varying doses of creatine were exposed to 200 μM of H_2_O_2_. A PCR-based assay was then used to assess the level of DNA damage in both systems, and HUVEC cell viability was further determined 72 h post-oxidative damage. Authors reported that creatine, in a dose-dependent manner, demonstrated the ability to protect both nDNA and mtDNA against oxidative damage; however, these protective benefits were more pronounced in HUVEC mtDNA than nDNA. Furthermore, HUVECs pretreated with creatine for 24 h showed increased cell viability in comparison to controls, and presented with significantly less mtDNA breaks and mutations. Guidi also concluded that creatine could play an important role in protecting vascular EC mtDNA against oxidative insults. Furthermore, Guidi proposed that creatine could aid in mitochondrial stability, contributing to the stabilization of oxidative phosphorylation and the reduction of mtROS production.

Although evidence suggests that creatine may protect DNA from detrimental damage, less is known regarding creatine’s impact upon RNA which is comparably susceptible to damage and similarly associated with vascular pathology risk. To address this, Fimognari et al. [116] aimed to investigate whether cultured Jurkat T-leukemia cell RNA could be protected against a variety of chemical insults, following either 1, 3 or 10 mM of creatine. The RNA toxic compounds utilized in this study included ethyl methanesulfonate (EMS), H_2_O_2_, doxorubicin (chemotherapy drug), spermine NONOate (a NO donor), and S-nitro-N-acetylpenicillamine (SNAP). The authors reported that all chemical insults significantly impaired cell vitality and damaged RNA. Creatine supplementation was found to significantly reduce RNA-damaging activity; however, was found to only do so with H_2_O_2_ and doxorubicin. Taking into consideration that H_2_O_2_ is a biologically prominent free radical, despite the inability to counteract other chemical insults, these results can still be considered promising. Fimognari concluded that creatine supplementation does indeed exhibit RNA-protective properties and hypothesized that this could have been partially due to its capacity to directly scavenge free radicals, and/or its ability to maintain cellular energy stores. Taking into consideration the detrimental role DNA and RNA damage plays in vascular pathologies [196,206], and the evidence that demonstrates the ability of creatine to protect and attenuate damage elicited by varying toxic stimuli, one could suggest that creatine may be a suitable therapeutic for the support of vascular health through the promotion of DNA and RNA maintenance and health. 

## 6. Discussion 

Creatine’s popularity as an ergogenic aid exponentially increased following the success of two notable Olympic gold medalists who consumed creatine supplementation during the 1992 Barcelona Olympic games [216,217]. Since its success on a global platform, the popularity of creatine has continued to grow, generating a substantial body of literature supporting its beneficial ergogenic effects, including improvements in lean mass [218], fatigue resistance [219], and intermittent high-intensity exercise capacity [220]. Furthermore, considering the physiological impairments such as intellectual disability, seizures, muscle weakness and gastrointestinal issues that symptomatically characterize genetic diseases of creatine deficiency (such as arginine: glycine amidinotransferase deficiency, guanidinoacetate methyltransferase deficiency, and creatine transporter deficiency); it is clear that creatine plays a vital role in bioenergetics, metabolism and overall cellular health. Due to its broad application and cellular importance, many have hypothesized the potential therapeutic role of creatine. Creatine supplementation has since been shown to benefit pathologies such as myopathies [60,61], chronic kidney disease [221], diabetes [57], and neurodegenerative disorders [63]. Research has additionally highlighted other non-energy related potentials of creatine, such as its ability to serve as both a direct- and indirect-antioxidant [52,53]. 

Due to the widespread potential of creatine, some have argued this supplement as one of the most promising, pleiotropic, nutritional supplement therapeutics currently available. Creatine can also be sourced, however, in its natural form. Arguably more beneficial in its whole food form due to the additional nutritional value, creatine concentrations can range from 3–5 g/kg of raw meat [47]. Despite these natural sources of creatine, to successfully ingest the recommended “loading” dose of 20 g/day required to rapidly increase skeletal muscle stores [48,49], one would have to consume approximately 4 kg of meat per day. Consuming this large amount of meat could be difficult for those with dietary restrictions, those conscious of calorie intake, those wanting cheaper alternatives, or those who are cautious of other macronutrients such as fat and protein. While skeletal muscle creatine stores can similarly be increased through the ingestion of smaller amounts (3–5 g/day) over longer durations of time [48], this would still require the consumption of 1 kg of meat a day, which may be unachievable for many. Thus, creatine supplementation in the zero-calorie (if unflavored), powdered monohydrate form specifically, has remained the most efficacious method of increasing dietary creatine intake. Furthermore, creatine supplementation has been found to exhibit an excellent safety profile. As covered by Kreider et al., clinical populations supplemented with high levels of creatine (0.3–0.8 g/kg/day or 21–56 g/day for a 70 kg individual) for up to 5 years, produced no reports of significant or severe adverse events [49]. This tolerance and safety profile has been reported in both young and older populations, over both acute and long-term periods [222]. The few common side effects mentioned in creatine literature include bloating and weight gain from increases in water retention [49], while serious adverse events such as kidney dysfunction are extremely rare and are often only a consequence of poor adherence or compliance with dosing recommendations. 

**Figure 1:**
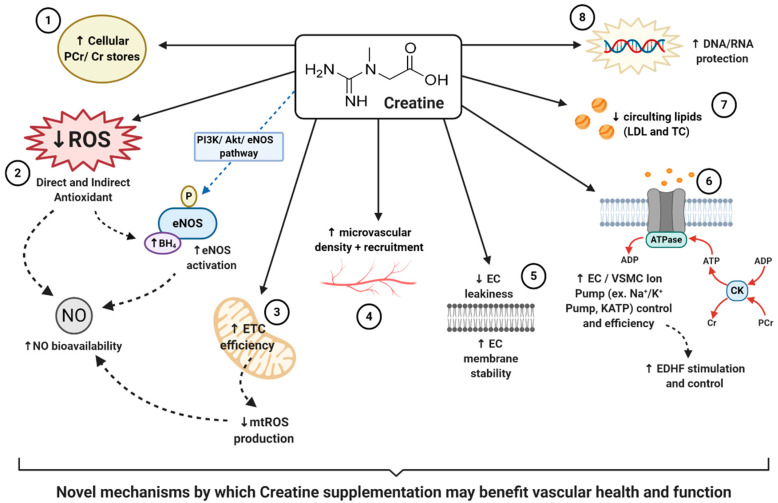
Novel mechanisms by which creatine supplementation may benefit vascular health and function; PCr = phosphocreatine, Cr = creatine, EC = endothelial cell, DNA = deoxyribonucleic acid, RNA = ribonucleic acid, ETC = mitochondria electron transport chain, mtROS = mitochondrial-specific reactive oxygen species, ROS = reactive oxygen species, LDL = low density lipoprotein, TC = total cholesterol, PI3K/Akt/eNOS pathway = Phosphatidylinositol 3-kinase/Protein kinase B/endothelial nitric oxide synthase pathway, eNOS = endothelial nitric oxide synthase, P = phosphorylation, BH_4_ = tetrahydrobiopterin, NO = nitric oxide, ADP = adenosine diphosphate, ATP = adenosine triphosphate, CK = creatine kinase, VSMC = vascular smooth muscle cell, Na^+^/K^+^ Pump = sodium/potassium pump, KATP = ATP-sensitive potassium pump, EDHF = endothelium-derived hyperpolarization factor, ↓ = decrease/reduction in, ↑ = increase in, “blue dashed line” = proposed pathway by Ahsan et al. [147], phosphorylation and stimulation of eNOS.

Although creatine supplementation appears to have high therapeutic potential, supporting published scientific literature is still limited in a variety of areas. For example, although creatine has shown great promise and has benefited certain clinical populations, there is scarce information on the role creatine plays within vascular health. Therefore, throughout this review we have discussed the few studies that have directly shown the potential of creatine supplementation to benefit vascular health. Furthermore, we have elucidated the novel ways in which creatine may serve to alleviate various risk factors that contribute to the development of vascular pathologies and CVDs. For example, we have touched upon the ability of creatine to (see Figure 1):

1. Increase natural EC stores of high-energy metabolites [54,90].
2. Serve as both a direct- and indirect- antioxidant [52,58,62,97], scavenging free radicals which could thereby improve eNOS efficiency, NO synthesis, and NO bioavailability.
3. Improve the integrity and efficiency of the mitochondria resulting in reduced mtROS production [58,215].
4. Increase microvascular density, recruitment, and vasomotor function [55,56].
5. Improve EC membrane stability and decrease EC leakiness [54,185,190,191].
6. Aid in the function of EC and VSMC energy-dependent ion pumps [176,177,178], thereby benefiting the propagation of EDHFs.
7. Reduce circulating amounts of damaging lipids such as LDL-C and total cholesterol [55,123].
8. Protect both DNA and RNA from cytotoxic stimuli such as oxidative stress [116,122,209,215].

In addition to these novel ways by which creatine may serve a protective role in vascular health, creatine has also been shown to help reduce inflammation and homocysteine; both of which are closely associated with incidence of CVD [68,69]. For more information regarding the impact of creatine on inflammation and homocysteine, readers are advised to reference the review by Clarke et al. [223]. Furthermore, evidence also suggests the potential of creatine and/or PCr to directly stimulate the synthesis of NO through the PI3K/Akt/eNOS pathway in endothelial cells [147]; however, this interesting mechanism requires further investigation.

## 7. Conclusions

To conclude, the current body of literature surrounding creatine’s more clinical applications, serving as a therapeutic or adjuvant therapy in human populations, is still relatively new and requires further investigation. Despite the availability of numerous in vitro and in vivo data supporting the potential of creatine to function in these new and novel ways, there is very little translational science investigating the impact of creatine supplementation on human vasculature, specifically. To date, only four studies have directly investigated the effects of creatine on the vasculature in humans. Therefore, as highlighted throughout this review, although there is evidence to suggest that creatine may possess unique properties that may impart novel benefits upon the vasculature, further clinical research is needed to truly determine the impact of creatine supplementation on vascular health. Furthermore, population specific clinical trials involving those at risk of, or presenting with, vascular pathology would further contribute to the body of knowledge, helping to uncover the potential applications of creatine supplementation within vascular health.

## Data Availability

No new data were created or analyzed by the authors and incorporated in this review. Data sharing is not applicable to this article.
